# Clinical Analysis of PRP Combined With Hyaluronic Acid Mesotherapy in the Treatment of Facial Chloasma

**DOI:** 10.1111/jocd.70286

**Published:** 2025-06-19

**Authors:** Yang Ling, Xinhao Liu, E. Yang

**Affiliations:** ^1^ Department of Dermatology The People's Hospital of Wansheng District Chongqing China; ^2^ Department of Burn and Plastic Surgery The First Affiliated Hospital of Chongqing Medical University Chongqing China

**Keywords:** chloasma, hyaluronic acid, mesotherapy, PRP

## Abstract

**Objective:**

To study the safety and clinical effect of PRP combined with hyaluronic acid (HA) mesotherapy in the treatment of facial chloasma, to explore a safe and effective treatment option for chloasma.

**Methods:**

57 patients with chloasma who received mesotherapy were analyzed in this study. They were divided into 19 cases of the PRP combined with HA mesotherapy group (PRP+HA group), 20 cases of the PRP mesotherapy group (PRP group) and 18 cases of the hyaluronic acid mesotherapy group (HA group). The therapeutic effect of chloasma was evaluated at the fourth week after treatment, including the decrease rate of chloasma area and severity index (MASI), the effective rate, the decrease rate of pigment degree, the decrease rate of pigment area, and the decrease rate of pigment quantity. The patient's satisfaction and adverse reactions were investigated. The safety and efficacy of each group were compared and analyzed.

**Results:**

The effective rate of chloasma treatment in the PRP group and the PRP+HA group was more than 50% at 4 weeks after treatment. The improvement of the above indexes in the PRP+HA group was better than those in the single treatment group. The satisfaction of the PRP+HA group was higher than that of the HA group, and the satisfaction of the other groups was not significantly different. There were no serious adverse reactions in the three groups.

**Conclusion:**

PRP mesotherapy alone or combined with HA and PRP mesotherapy can effectively treat chloasma, and the combined treatment effect is better, which is worthy of clinical promotion.

## 1 Introduction

Chloasma is a common, chronic, pigmented skin disease characterized by light brown or dark brown spots and patches with different depths and unclear boundaries on the face. The incidence of chloasma in Asian women of childbearing age is as high as 30% [1]. Genetic susceptibility, sunlight exposure, and changes in sex hormone levels are the main pathogenic factors of chloasma. Increased melanin synthesis, vascular proliferation at skin lesions, inflammatory response, and skin barrier damage are all involved in the occurrence of chloasma. Although there are many kinds of treatment methods for chloasma, the unsatisfactory treatment effect and recurrence after treatment are still a problem for the people [2]. In recent years, platelet‐rich plasma (PRP) has been used in the field of plastic surgery and has achieved good results in the treatment of chloasma, inflammatory pigmentation, acne, and facial skin photoaging [3]. PRP is rich in a variety of growth factors, among which TGF‐β can reduce tyrosinase and tyrosinase‐related proteins and play a major role in melanin synthesis, thus it can help treat pigmentation in chloasma [4]. Hyaluronic acid (HA), as the main component of the extracellular matrix, is mainly present in the skin and plays an important role in skin metabolism, hydration, maintenance of tissue structure and function, and cell proliferation, differentiation and migration [5]. HA is the main component to maintain epidermal hydration and has the function of repairing the epidermal barrier. HA can regulate the water balance in the tissue through its hydration, promote the excretion of metabolic waste and pigment, and increase the elasticity of the skin by promoting the secretion of fibroblasts and synthesizing collagen and elastic fibers, so as to effectively treat the coarse pores and promote facial rejuvenation [6]. However, there is no report on the use of HA alone in the treatment of chloasma. Mesotherapy can quantitatively inject drugs into the specific dermis, so that the active ingredients directly act on the skin and play a corresponding role. Its acupuncture reaction can also cause controllable damage to the skin. This micro‐damage can lead to subtle rupture of blood vessels under the epidermis, release a variety of growth factors, promote the production of collagen and elastin, and thus achieve skin rejuvenation [7].

We use HA combined with PRP Mesotherapy for facial rejuvenation to leverage the synergistic benefits of both agents and use mesotherapy to directly transport the active ingredients to the skin dermis, hoping to get better results.

## 2 Methods

### 2.1 Clinical Information

From October 2023 to June 2024, 60 patients with chloasma who volunteered to receive mesotherapy were recruited and randomly divided into PRP+HA group, PRP group, and HA group by random number method, with 20 cases in each group. Inclusion criteria: (1) Aged 18–60 years (2) Diagnosed with facial chloasma by at least two dermatology specialists and willing to accept treatment; (3) Voluntary participation in this study and signed informed consent; (4) Willing to regular follow‐up observation. Exclusion criteria: (1) Received facial treatment within the past 2 months (2) During the study period, the application of other cosmetic treatments affected the judgment of the results; (3) Pregnant or lactating women; (4) Psychiatric patients, alcoholics, drug users; (5) Patients with autoimmune diseases; (6) Patients with a history of severe skin allergy; (7) Patients with a history of HA or lidocaine allergy; (8) Coagulation dysfunction.

### 2.2 Treatment

The preparation before treatment included preoperative communication, signing informed consent, random grouping, cleaning facial skin, collecting general photography, and skin imaging analyzer to detect skin pigmentation. After 30 min of anesthesia with lidocaine cream, the face was disinfected, and the mesotherapy began.

PRP+HA group: 20 mL venous blood was extracted from the patients to prepare 2.5 mL PRP; PRP was prepared by blood component separation machine(Sichuan Nigale Biomedical Co. Ltd.), and 2.5 mL non‐crosslinked HA mixed into 5 mL mixture. PRP group: 20 mL venous blood was extracted from the patients to prepare 2.5 mL PRP, and 2.5 mL normal saline mixed into 5 mL mixture. HA group: 2.5 mL of non‐crosslinked HA and 2.5 mL of normal saline mixed into 5 mL mixture. All three groups were treated with the same method of mesotherapy machine for comprehensive intradermal injection. After the injection, the face was cleaned, and the medical wound dressing mask (Sichuan Santai Pharmaceutical Technology Co. Ltd) was applied for 15 min.

**FIGURE 1:**
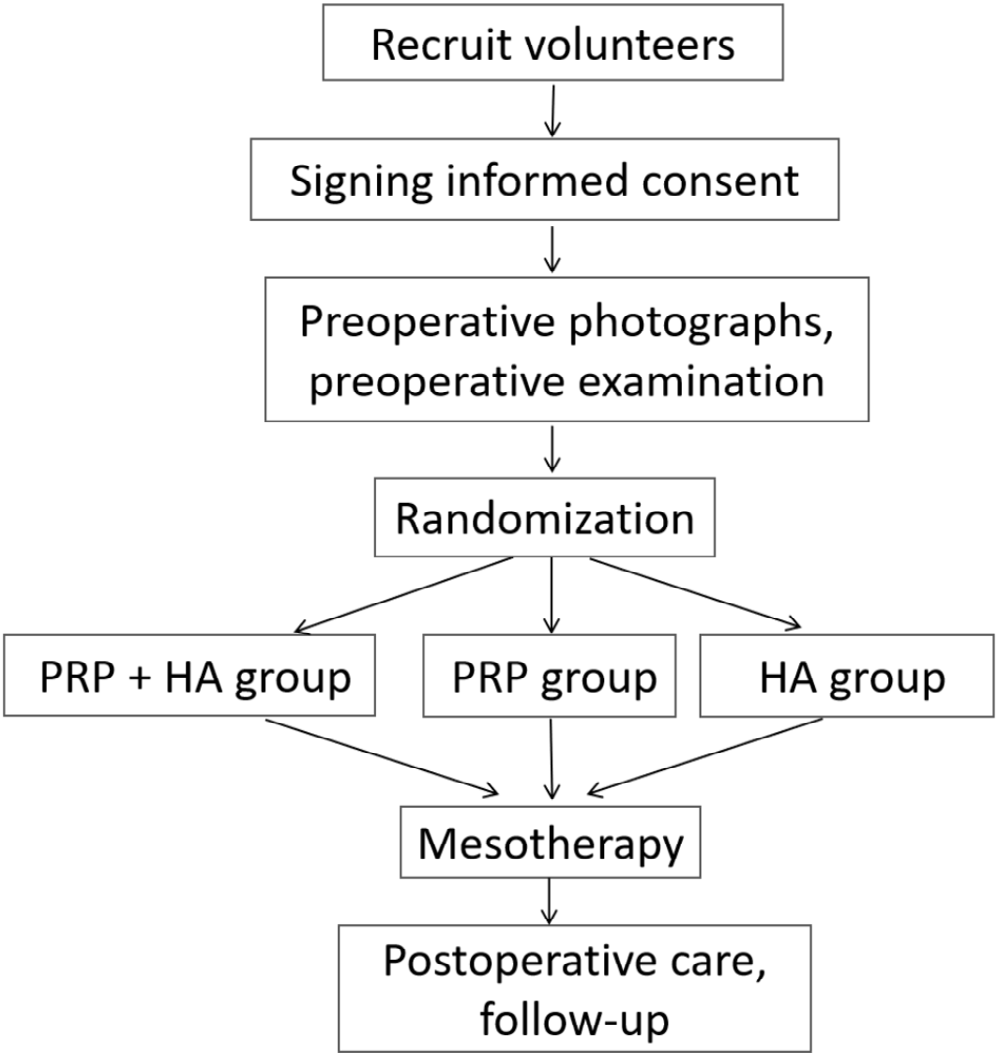
Patient grouping flow chart.

Postoperative care: Normal saline can be used to wipe the facial exudate within 24 h after treatment. After 24 h, the patient could wash his or her face and skin normally. Within 1 week, the sterile medical wound dressing mask was applied once a day. Strong light irradiation was avoided within 1 month. Patients need to return to the hospital for follow‐up 4 weeks after treatment (Figure 1).

### 2.3 Safety Assessment

Adverse events during and after treatment were observed and recorded, including local adverse reactions (bruising, redness, swelling, pain, tenderness, and itching) and systemic adverse reactions.

### 2.4 Evaluation of Efficacy

The evaluation was conducted before treatment and 4 weeks after treatment. The evaluation indicators include:

1. Chloasma area and severity index (MASI)

Two specialists with more than 10 years of work were selected as fixed judges to evaluate and record the skin lesions of chloasma according to MASI. The treatment plan was blind to the evaluators.

MASI standard: quantitative according to the area, color depth and color uniformity of chloasma. Evaluation of pigmentation area: 30% of the forehead (F), 30% of the right cheek (MR), 30% of the left cheek (ML), and 10% of the mandible (C) were evaluated. According to the proportion of pigment spots in these four regions, the scores (A) were calculated as follows: < 10% was recorded as 1 point, 10%–29% was recorded as 2 points, 30%–49% was recorded as 3 points, 50%–69% was recorded as 4 points, 70%–89% was recorded as 5 points, 90%–100% was recorded as 6 points. Color depth (D) and uniformity (H) score: 0 ~ 4 points: 0 is no, 1 is slight, 2 is moderate, 3 is obvious, 4 is the maximum.

MASI = forehead [0.3A (D + H)] + right cheek [0.3A (D + H)] + left cheek [0.3A (D + H)] + mandible [0.1A (D + H)]. The maximum is 48 points, the minimum is 0.

MASI decline rate (%) = (total score before treatment‐total score after treatment)/total score before treatment × 100%. Efficacy evaluation criteria: invalid refers to the MASI decline rate < 19%, effective refers to the MASI decline rate 20% ~ 59%, markedly effective refers to the MASI decline rate 60% ~ 89%, basic recovery refers to the MASI decline rate ≥ 90%. The total effective rate was calculated as (%) = (basic recovery + markedly effective + effective)/total cases × 100%.

2. Patient satisfaction evaluation

Through the form of questionnaire, the patient ‘s satisfaction with the curative effect was investigated, which was divided into: 5 points as very satisfied; 4 points as satisfactory; 3 points as general; 2 points as dissatisfied; 1 point as very dissatisfied.

3. Image analysis of chloasma pigment

The photos of emmetropia, left side and right side were recorded by skin image analyzer (ISEMECO Skin Imaging Analyzer). The degree of pigmentation, pigment area, and pigment quantity of facial skin were recorded. The values were obtained by the skin imaging analyzer. We photographed and analyzed the pigmentation spots before and 4 weeks after the operation. The improvement at the fourth week after treatment was calculated = (pre‐treatment data‐post‐treatment data)/pre‐treatment data × 100%.

### 2.5 Statistical Analysis

SPSS 25.0 software was used to analyze the differences in the effects of each group before and after treatment. The measurement data conforming to the normal distribution were expressed as $\bar{x}$ ± s. One‐way analysis of variance was used for comparison among multiple groups. Post hoc analyses were conducted where appropriate using Tukey's test. *p* < 0.05 indicated that the difference was statistically significant.

## 3 Results

### 3.1 Baseline Clinical Data

A total of 60 people were enrolled, 20 people in each group, including 1 lost in the PRP+HA group and 2 lost in the HA group. A total of 57 people completed the follow‐up and were included in the statistics. There was no significant difference in the baseline data of age, MASI, degree of chloasma pigmentation, area, and quantity between the groups (*p* > 0.05), which was comparable (Table 1).

**TABLE 1:** Baseline characteristics of study participants.

PRP+HA group		PRP group	HA group	*p* value		
				PRP+HA vs. PRP	PRP+HA vs. HA	PRP vs. HA
MASI	6.347 ± 3.029	7.035 ± 2.841	7.311 ± 2.071	0.429	0.281	0.753
Age	39.11 ± 6.523	39.90 ± 7.078	37.72 ± 6.632	0.715	0.536	0.326
Pigment degree	42.17% ± 8.47%	39.98% ± 7.66%	38.85% ± 9.24%	0.423	0.238	0.683
Pigment area	10.59% ± 3.49%	8.73% ± 3.91%	9.33% ± 1.87%	0.079	0.242	0.574
Pigment number	414.00 ± 105.62	363.15 ± 119.31	393.22 ± 61.68	0.117	0.529	0.357

### 3.2 Efficacy Evaluation

The effective rate of MASI in the PRP+HA group was 100%, the effective rate in the PRP group was 70%, and the effective rate in the HA group was 22.22%. The decrease rate of MASI in the PRP+HA group was 42.88% ± 13.71%, and the PRP+HA group showed significantly greater MASI score reduction compared to both the PRP(28.22% ± 14.32%) and HA groups (14.42% ± 11.34%). The difference between the groups was statistically significant (*p* < 0.05).

**FIGURE 2:**
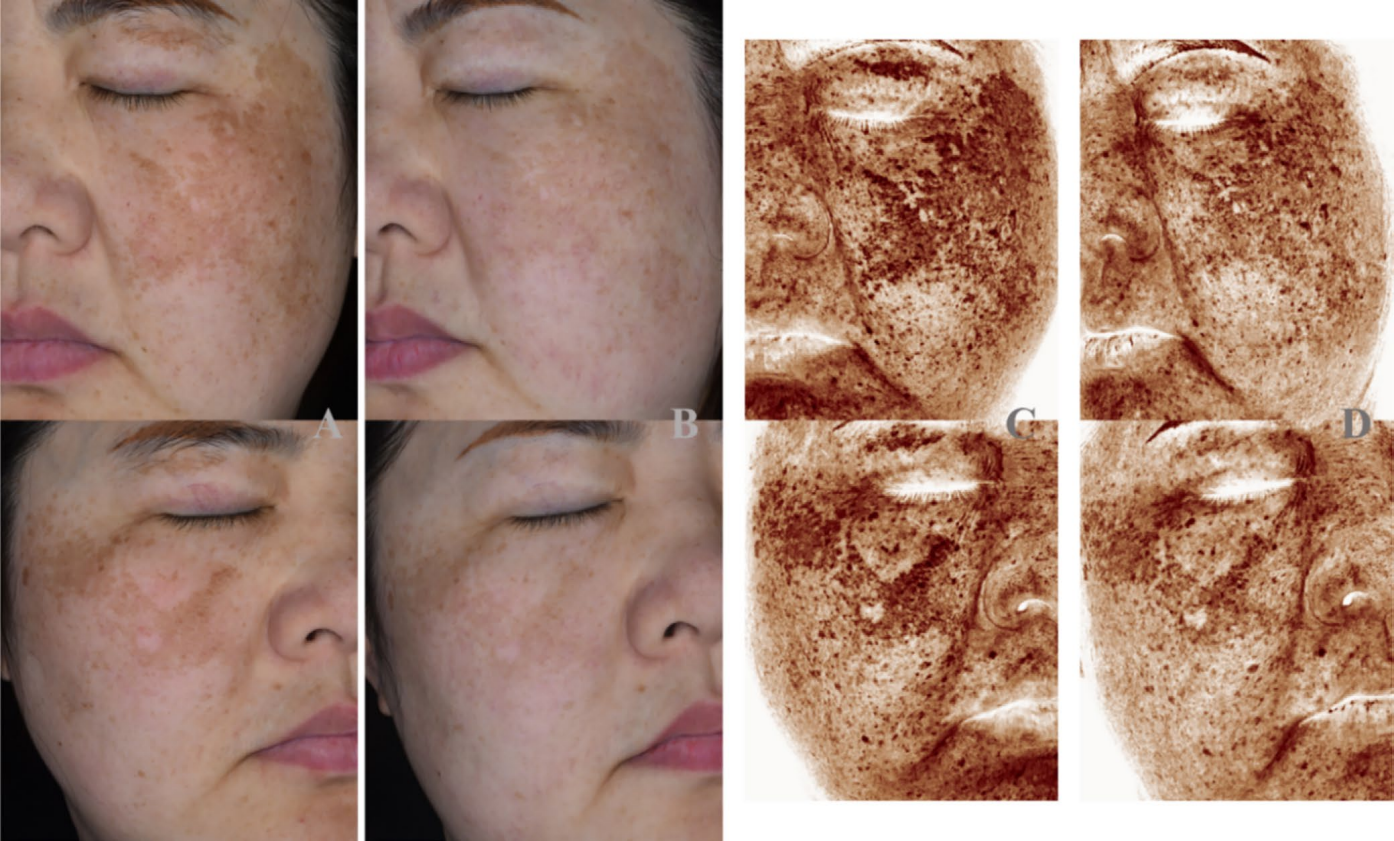
Comparison of PRP combined with hyaluronic acid mesotherapy before and after treatment. (A) Photos before treatment; (B) 4 weeks after treatment photos; (C) Pigment before treatment; (D) Pigment at 4 weeks after treatment)

Before treatment and 4 weeks after treatment, the reduction rates of skin pigment degree, pigment area, and pigment number were detected and analyzed by skin image analyzer. The combined treatment can better reduce the degree of pigment (Figure 2). The reduction rates of pigment degree in the PRP+HA group, PRP group, and HA group were 27.55% ± 6.88%, 19.05% ± 6.46%, and 8.49% ± 9.09%, respectively. The difference between the groups was statistically significant (*p* < 0.05). The reduction rates of pigment area in the PRP+HA group, PRP group, and HA group were 19.06% ± 13.85%, 12.27% ± 13.65%, and 7.87% ± 7.61%, respectively. There was a statistically significant difference between the PRP+HA group and the HA group (*p* < 0.05), but there was no significant difference between the other two groups (*p* > 0.05). The reduction rates of pigment number in the PRP+HA group, PRP group, and HA group were 14.73% ± 7.31%, 10.79% ± 11.55%, and 5.76% ± 6.83%, respectively. There was a statistical difference between the PRP+HA group and the HA group (*p* < 0.05), but there was no significant difference between the other two groups (*p* > 0.05). The satisfaction of the PRP+HA group was higher than that of the HA group (*p* < 0.05), but there was no significant difference in satisfaction between the other groups (*p* > 0.05) (Table 2). All the three groups had slight swelling and slight congestion around the eyes after treatment, and no patients had serious adverse reactions and complications.

**TABLE 2:** Comparison of pigment reduction and satisfaction between groups.

	PRP+HA group	PRP group	HA group	*p* value		
				PRP+HA vs. prp	PRP+HA vs. HA	PRP vs. HA
MASI decline rate	42.88% ± 13.71%	28.22% ± 14.32%	14.42% ± 11.34%	***< 0.05***	***< 0.05***	***< 0.05***
Pigment degree reduction rate	27.55% ± 6.88%	19.05% ± 6.46%	8.49% ± 9.09	***< 0.05***	***< 0.05***	***< 0.05***
Pigment area reduction rate	19.06% ± 13.85%	12.27% ± 13.65%	7.87% ± 7.61%	0.087	***< 0.05***	0.270
Pigment number reduction rate	14.73% ± 7.31%	10.79% ± 11.55%	5.76% ± 6.83%	0.173	***< 0.05***	0.088
Satisfaction score	3.26 ± 0.65	2.85 ± 0.67	2.61 ± 0.70	0.061	***< 0.05***	0.280

*Note:* Bold and italics values indicated that the difference was statistically significant.

## 4 Discussion

### 4.1 Recap Pathogenesis of Chloasma

Chloasma is a chronic true epidermal hyperpigmentation skin disease with high incidence, poor response to treatment, and high recurrence rate, which has caused a great psychological burden to many patients. It is believed that in addition to the increase of pigment granules in the epidermis, skin barrier function and structural disorders, skin barrier structure and dysfunction, up‐regulation of inflammatory factor expression, high oxidative stress, increased mast cells, vascular proliferation, basement membrane zone, and dermal matrix damage lead to the abnormal morphology of melanocytes and other changes that are involved in the occurrence and development of chloasma [8]. Although there are many treatment options for chloasma, conventional treatment is difficult to achieve the ideal state or complete improvement. Therefore, the treatment of chloasma has always been challenging in the field of medical cosmetology, and further exploration of safer and more effective alternative treatment options is still needed.

### 4.2 Mechanisms of PRP and HA

PRP is rich in a variety of growth factors and elastic fibers, protein micro fibers, endogenous micro fibers [9]. The pH of PRP is 6.5–6.7, and the weak acid environment can inhibit the growth of bacteria and promote the secretion of a large number of growth factors. PRP can trigger tissue regeneration procedures, stimulate the proliferation and differentiation of stem cells to activate fibroblasts in the dermis, promote the synthesis of collagen, HA, and other matrix components, provide skin tension and elasticity, and play a role in skin rejuvenation [10]. The effective factors in PRP can competitively bind to the active center of tyrosinase to reduce the production of dopa. It can also inactivate tyrosinase by chelating copper ions, inhibit melanin formation, and reduce pigmentation [11]. After activation, growth factors (GFs) in platelet α granules, combined with other chemokines and cytokines, can be used as a regulatory signal to improve chloasma by inhibiting melanin production, repairing the skin physiological barrier, promoting the normalization of true epidermal structure, and up‐regulating matrix gene expression. It is considered to be the basis for PRP to be applied to chloasma and achieve clinical improvement [12]. For example, TGF‐β can inhibit the expression of the PAX3 gene in melanocytes and down‐regulate the promoter activity of microphthalmia‐associated transcription factor (MITF) in a concentration‐dependent manner and reduce the production of tyrosinase‐related proteins at the protein level [13]. EGF could inhibit the expression of prostaglandin E2 and the activity of tyrosinase [14]. PDGF can increase the volume of the dermis, including the normalization of blood vessels, collagen, and extracellular matrix, and thus increase the skin glossiness [15]. In recent years, several studies have attempted to apply PRP to the treatment of chloasma and achieved certain clinical results [4, 16, 17, 18]. The effect of PRP intradermal injection combined with oral tranexamic acid in the treatment of chloasma is better than that of PRP alone [19].

HA is one of the main components of the skin dermis. It plays an important role in skin metabolism, hydration, maintenance of tissue structure and function, cell proliferation, differentiation, and migration. It has a strong moisturizing effect [20]. Intradermal injection of HA through mesotherapy can effectively maintain skin moisture, promote cell activity, and accelerate local pigment metabolism. HA can reduce the damage of oxygen free radicals to cell structure and promote skin barrier repair by inhibiting the formation of oxygen free radicals in cells, which is conducive to improving the severity of chloasma [21]. In addition to the quantitative injection of drugs into the specific dermis, the acupuncture reaction of mesotherapy can also cause controllable damage to the skin. This micro‐damage can lead to subtle rupture of blood vessels under the epidermis, release a variety of growth factors, promote the production of collagen and elastin, and thus achieve skin rejuvenation [22].

### 4.3 Interpretation of Study Results, Comparison to Literature, Limitations, Future Directions

It has been reported that PRP mesotherapy can effectively treat chloasma. And HA mesotherapy can increase skin elasticity, so as to effectively improve the coarse pores and achieve facial rejuvenation. In our study, PRP combined with HA mesotherapy was used to treat chloasma. The advantages of PRP and HA were combined to promote skin pigment metabolism, supplement skin moisture, and further reduce the pigment to treat chloasma. The treatment can inject the substance into the dermis of the skin more accurately and evenly by means of mesotherapy to make it work. Previous studies have mostly used PRP alone or HA alone or PRP combined with other drugs to treat chloasma, and there are few reports of PRP combined with HA in the treatment of chloasma. This study shows that the combination of PRP and HA mesotherapy is indeed better than PRP alone or HA alone in the treatment of chloasma pigmentation. Although we only conducted one time treatment study analysis, the combined treatment group has obvious advantages. One treatment has shown obvious advantages in desalinating the pigment of chloasma. We hypothesize that repeated treatments may yield enhanced and more sustained results. However, there was no significant advantage in the reduction of pigment area and pigment number in a single combined treatment. The treatment of chloasma is a long‐term and complex work. The pigment needs to be slowly desalinated before it disappears. Our research has only reached the degree of pigment fading, and has not yet reached the disappearance of pigment. Therefore, the pigment area and pigment number did not show significant changes. We believe that multiple treatments will have a better effect. We will also further explore the best treatment course and cycle in the next study.

## Author Contributions

**Yang Ling:** implement research work, drafting the work, final approval of the version to be published, agreement to be accountable for all aspects of the work in ensuring that questions related to the accuracy or integrity of any part of the work are appropriately investigated and resolved. **Xinhao Liu:** data analysis, follow‐up subjects and article writing. Final approval of the version to be published, agreement to be accountable for all aspects of the work in ensuring that questions related to the accuracy or integrity of any part of the work are appropriately investigated and resolved. **E. Yang:** design research content, analysis data for the work, revising important intellectual content, approval of the version to be published, agreement to be accountable for all aspects of the work in ensuring that questions related to the accuracy or integrity of any part of the work are appropriately investigated and resolved.

## Disclosure

The authors have nothing to report.

## Ethics Statement

The authors have nothing to report.

## Conflicts of Interest

The authors declare no conflicts of interest.

## Data Availability

The data that support the findings of this study are available on request from the corresponding author. The data are not publicly available due to privacy or ethical restrictions.
